# Prevalence and correlates of non-adherence to immunosuppressants and to health behaviours in patients after kidney transplantation in Brazil – the ADHERE BRAZIL multicentre study: a cross-sectional study protocol

**DOI:** 10.1186/s12882-018-0840-6

**Published:** 2018-02-20

**Authors:** Helady Sanders-Pinheiro, Fernando Antonio Basile Colugnati, Elisa Oliveira Marsicano, Sabina De Geest, José Osmar Pestana Medina, on behalf of Adhere Brazil Consortium Group, Bartira A. Roza, Samira S. Almeida, Marilda Mazzali, Helio Tedesco-Silva, Paula F.C.B.C. Fernandes

**Affiliations:** 1 Renal Transplantation Unit, University Hospital, Federal University of Juiz de Fora, Rua Benjamin Constant, 1044/1001, Juiz de Fora, MG 36015-400 Brazil; 2 Núcleo Interdisciplinar de Estudos e Pesquisas em Nefrologia [NIEPEN], Rua Benjamin Constant, 1044/1001, Juiz de Fora, MG 36015-400 Brazil; 3 Institute of Nursing Science, Department of Public Health, Faculty of Medicine, University of Basel, Basel, Switzerland; 4 Academic Centre for Nursing and Midwifery, Department of Public Health and Primary Care, Faculty of Medicine, KU-Leuven, Leuven, Belgium; 5 Hospital do Rim e Hipertensão, Federal University of São Paulo, São Paulo, Brazil

**Keywords:** Patient adherence, Medication nonadherence, Immunosuppression, Health behaviour, Kidney transplantation, Brazil, Design, Healthcare system

## Abstract

**Background:**

Non-adherence to immunosuppressive therapy is a prevalent risk factor for poor clinical and after kidney transplantation (KT), and has contributed to the lack of improvement in long-term graft survival over the past decade. Understanding the multilevel correlates and risk factors of non-adherence is crucial to determine the optimal level for planning interventions, namely at the patient, health care provider, KT centre, and health care system level. Brazil, having the largest public transplantation program in the world and with regional differences regarding access to health services and service implementation, is in a unique position to study this multilevel approach. Therefore, the Adhere Brazil Study (ADHERE BRAZIL) was designed to assess the prevalence and variability of non-adherence to immunosuppressants and to health behaviours among adult KT recipients in Brazil, and to assess the multilevel correlates of non-adherence to immunosuppressive medication. We describe the rationale, design, and methodology of the ADHERE BRAZIL study.

**Methods/Design:**

This is an observational, cross-sectional, multicentre study that includes 20 Brazilian KT centres. A stratified sampling approach is used, based on strata, with the following characteristics considered: geographical region and transplant activity (number of KTs per year). A random sample of patients (proportional to the size of the centre within each stratum) is selected from each centre. The prevalence of different health behaviours is assessed through self-report. The assessment of multilevel correlates of non-adherence is guided by the ecological model that considers factors at the level of the patient, health-care professional, and transplant centre, using established instruments or instruments developed for this study. Data will be collected over an 18-month period, with information obtained during the regular follow-up visits to the transplant outpatient clinic and directly entered into the Research Electronic Data Capture (RedCap) system. Data entry is performed by a trained professional who is part of the transplant team. The data collection began in December 2015.

**Discussion:**

This multicentre study is the first to evaluate multilevel correlates of non-adherence in KT patients and will provide a reliable estimate of non-adherence in Brazilian KT patients.

**Trial registration:**

ClinicalTrials.gov on 10/10/2013, NCT02066935.

**Electronic supplementary material:**

The online version of this article (10.1186/s12882-018-0840-6) contains supplementary material, which is available to authorized users.

## Background

Kidney transplantation (KT) is the renal replacement therapy that provides a better quality of life and longer survival [1, 2]. However, the long-term graft outcome has not improved over the last decade, and remains an important clinical problem [3, 4]. Non-adherence to immunosuppressive therapy is a prevalent risk factor for this worse outcome in KT and consequently contributes to the lack of improvement in the long-term graft survival [5–7]. Assessing the correlates and risk factors of non-adherence is crucial to guide intervention development, i.e. interventions needed at patient, health care providers, KT centres or health care system level [5, 6, 8, 9].

**Fig. 1:**
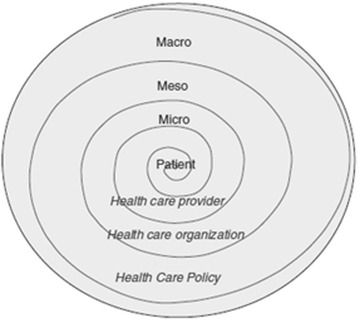
Ecological model used, adapted from Bronfenbrenner et al., [12] and Berben, [9] (with permission)

The World Health Organization defines adherence as a multidimensional process that involves factors related to the following: socioeconomic profile (family income, social support, cost of medications), the characteristics of the disease and its treatment (comorbidities, chronicity of the disease, complexity of regimen), the patient as an individual (self-efficacy, health beliefs, health literacy), the health care professionals involved in the treatment, and the health care system [10]. However, in transplantation, as in other chronic diseases, most studies have focused only on the socioeconomic, disease- and treatment-related factors [5, 8, 10, 11]. A meta-analysis of 29,000 KT patients, performed in the modern era of immunosuppressive therapy, reported that non-adherence was explained only in part by the factors included in the study. The authors suggested that other factors, potentially linked to higher levels of care (i.e. health care team organization and health care system), also needed to be considered in order to understand patient non-adherence [11]. This view is reflected by the ecological model shown in Fig. 1, which posits that a patient’s behaviour results from multilayered influences at the level of the health care provider (micro), health care organization (meso) and health care system and policies (macro) [9, 12]. For instance, it has been shown that practice patterns that apply better principles of chronic illness management (CIM) are associated with favourable clinical and health care utilization outcomes in chronically ill populations [13]. Two studies had found similar results in KT patients [14, 15]. KT can be defined as a chronic disease as transplant patients are dependent on lifelong therapy and follow-up. Moreover, KT requires active patient participation in specific health behaviours to improve outcomes, such as: medication taking, maintenance of regular physical activity, reduction/cessation of alcohol intake, protection from ultraviolet sun, cessation of smoking or maintenance of a non-smoking status, and keeping medical appointments [16, 17].

To date, only one study has comprehensively addressed the multilevel factors associated with non-adherence to immunosuppressive drugs among 1680 heart transplantation recipients, in 36 centres across 11 countries and 4 continents - The BRIGHT Study (Building Research Initiative Group: Chronic Illness Management and Adherence in Transplantation). The BRIGHT study also mapped practice patterns related to CIM in heart transplantation [18, 19]. Such elaborate data is lacking for KT and could contribute to identify leveraging points for adherence interventions.

Our group has recently evaluated selected multilevel correlates in a single centre study involving patients after the first year post-KT, in Brazil. Non-adherence to immunosuppressive therapy was identified in half of the patients. Surprisingly, a better economic profile, reflected by a higher family income, was the only variable that correlated to non-adherence. Given the inclusion of a single centre, meso level factors, such as practice patterns during post-transplantation follow-up, could not be evaluated [20].

Although non-adherence is a frequently reported behaviour in KT recipients, its prevalence varies according to the method of diagnosis, operational definitions used, and sampling method [5, 6, 11]. Adherence is defined as an agreement between a patient’s behaviour and the prescribed treatment, including the extent to which a patient takes medications, follows a diet, and/or implements lifestyle changes as prescribed by health care providers [10]. In transplantation, while studies have largely focused on adherence to immunosuppressive therapy [21], less attention has been given to adherence to other health behaviours [11]. The prevalence of non-adherence to prescribed immunosuppressive regimen among KT recipients ranges between 19 and 25 cases per 100 patients per year (PPY) [11]. Consequences of non-adherence are serious, including more acute rejection episodes, worse graft function and reduced survival, greater morbidity, and greater costs to the health systems [5, 6, 8, 22–26]. In the absence of a gold standard method to assess non-adherence to immunosuppressive therapy, a combination of methods is preferred to maximize the sensitivity of the assessment [27]. However, only a few studies have evaluated adherence to immunosuppressive therapy applying this methodology [20, 28, 29].

Adherence to other health behaviours after KT has been much less studied. The prevalence of smoking ranges from 2.8 to 4.0 PPY (median, 3.4 PPY) [11], and has been associated with cardiovascular diseases and higher mortality [30]. Less than two KT patients per 100 PPY are reported as not limiting/quitting alcohol consumption [11], but it is not clear if alcohol consumption is a risk factor for worse allograft and patient survival [31, 32]. Non-adherence to physical activity is prevalent, at 21.8 cases per 100 PPY [11, 33, 34]. Exercise interventions showed positive effects on intermediate outcomes, such as higher aerobic capacity and muscle strength; however, strong evidences on improved mortality and graft survival do not exist [34, 35]. One study reported non-adherence to medical appointments to be low among KT patients, at 4.7 cases per 100 PPY, with no association with worse outcomes [11].

Brazil is in a unique position with regard to KT, ranking second worldwide, in the absolute number of KTs performed and having the largest public transplantation program. However, regional discrepancies in transplant activity exist in Brazil, due to differences in regional population density, human development index, and the number of transplant services and trained professionals available. The largest proportion of KT activity is concentrated in the South and Southeast regions of the country and in large transplant centres with high transplant activity [36–39]. These regional differences, within the same country and under the same health care system, provide a valuable opportunity to explore how differences in service implementation and access to health services can influence non-adherence after KT. To date, only a few Brazilian studies have evaluated the prevalence of non-adherence to immunosuppressive therapy and health behaviours after KT [20, 40–47].

The ADHERE BRAZIL study aims to explore these gaps, specifically the prevalence of non-adherence to different health behaviours in the Brazilian KT population, considering the potential differences in access and challenges of the Brazilian Health Care System and also performing a broad evaluation of multilevel correlates to non-adherence to immunosuppressive therapy, including factors at the level of patients, health care providers, health care services, and health policies. Involving centres from all national regions and with different patterns of transplant activity will provide us with the valuable opportunity to benchmark patient non-adherence to KT treatment, characterize differences in patterns of clinical practice that may be associated with non-adherence, and a rare opportunity to share experiences among centres.

## Methods/Design

The aims of the ADHERE BRAZIL study are as follows: 1. To estimate the prevalence of non-adherence to immunosuppressants, and to other treatment-related aspects (smoking cessation, alcohol consumption, physical activity, and appointment keeping), in KT recipients among different KT centres across different regions of Brazil; 2. To explore multilevel factors associated to immunosuppressive adherence at the level of patient (socio-demographic, clinical), healthcare provider (patient satisfaction with the interpersonal dimension of care, trust in the transplant team, social support), healthcare organization (composition of the team, operational access, CIM transplant program practice patterns), and healthcare system and policies (perceived financial burden of the treatment regimen, insurance status, barriers to access to the immunosuppressive drugs, Brazilian region); and 3. To benchmark the participating centres, regarding their practice patterns that are associated with non-adherence to health behaviours after KT.

### Study design

The ADHERE BRAZIL study is a multicentre, national, cross-sectional, observational study, based on survey design. The methodology is derived from the BRIGHT study [18].

### Sampling design and setting

**Fig. 2:**
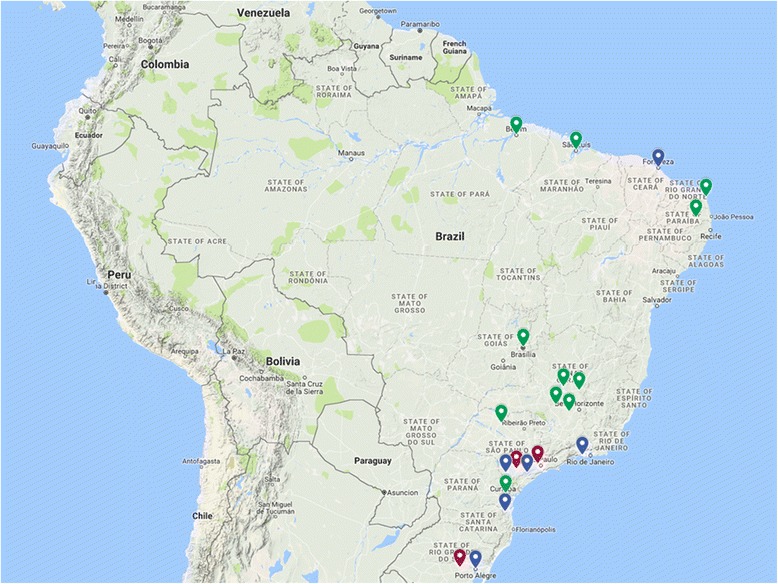
Location of centres participating in the study. Transplant activity is indicated in colour, as follows: Red, high activity (> 150 kidney KTs/year); green, moderate activity (50 to 150 KTs/year); blue, low activity (< 50 KTs/year)

A convenience sample of 20 centres was identified for inclusion, using a stratified sampling strategy. In Brazil, the KT activity of a centre is strongly associated with its geographical regions and its regional economic development. High activity centres are concentrated in the South and Southeast regions of the country, with low/moderate activity centres being more prevalent in the North, Northeast, and Midwest regions. To ensure an adequate representativeness from the enrolled centres, we defined four strata of equal size (approximately 283 patients): North, Northeast and Midwest with low or moderate activity; South and Southeast with low activity; South and Southeast with moderate activity; and South and Southeast with high activity. In each stratum, the number of patients in each centre is proportionally defined by the number of patients being followed up. The inclusion criteria for centres were as follows: performance of ≥10 KTs per year over the 5-year period preceding the study (2010-2014) and a signed agreement provided by the centre’s director. The 20 centres participating in the study are listed in Table 1, with their geographical distribution shown in Fig. 2. The majority of centres were located along the coast, following the same distribution as large- and medium-sized Brazilian cities [48].

**Table 1:** Participating centres of the ADHERE BRAZIL Study, divided by Brazilian geographical regions and transplant activity

Centre	Brazilian region	Transplant activity ^a^
Hospital Ophir Loyola – Belém/PA	North	Low
Hospital Universitário do Maranhão – São Luiz/MA	Northeast	Low
Hospital Antônio Targino Ltda. - Campina Grande/PB	Northeast	Low
Hospital Universitário Onofre Lopes - Natal/RN	Northeast	Low
Hospital Universitário Walter Cantídio – Fortaleza/CE	Northeast	Moderate
Hospital Universitário de Brasília – Brasília/DF	Midwest	Low
Centro Estadual de Transplantes/Hospital São Francisco de Assis na Providência de Deus - Rio de Janeiro/RJ	Southeast	Moderate
Fundação IMEPEN/Hospital Universitário da Universidade Federal de Juiz de Fora – Juiz de Fora/MG	Southeast	Low
Fundação Osvaldo Ramos - Hospital do Rim e Hipertensão/UNIFESP – São Paulo/SP	Southeast	High
Hospital Israelita Albert Einstein – São Paulo/SP	Southeast	Moderate
Hospital São João de Deus – Divinópolis/MG	Southeast	Low
Hospital das Clínicas da Universidade Estadual de Campinas/Unicamp – SP	Southeast	Moderate
Hospital das Clínicas de São Paulo – São Paulo/SP	Southeast	High
Instituto de Urologia e Nefrologia - Hospital de Base São José Rio Preto – São José do Rio Preto/SP	Southeast	Low
Santa Casa de Misericórdia de Belo Horizonte – Belo Horizonte/MG	Southeast	Low
Santa Casa de Misericórdia de Juiz de Fora – Juiz de Fora/MG	Southeast	Low
Fundação Pró Rim/Hospital Municipal São José – Joinvile/SC	South	Moderate
Hospital Angelina Caron – Curitiba/PR	South	Low
Hospital das Clínicas de Porto Alegre – Porto Alegre/RS	South	High
Santa Casa de Porto Alegre – Porto Alegre/RS	South	High

^a^ Volume of transplants performed: low activity, < 50 KTs/year; moderate activity, 50 to 150 KTs/year; and high activity ≥150 KTs/year

KT patients are randomly selected using a computer-generated list (Blockrand package for R language) and screened according to the following inclusion criteria: recipient of single and first KT; age > 18 years; > 1-year post-transplant; and ability to understand the objectives of the study. Patients are excluded if the immunosuppressive regimen is only based on drugs for which the blood monitoring is not available or not covered by Brazil’s health care system, e.g. mycophenolates without calcineurin or proliferation signal inhibitors. Patients randomly selected and meeting our inclusion criteria are invited to participate during their routine medical consultation visits, and those providing written informed consent are enrolled.

Using the OpenEpi stats program, the sample size was calculated for studies of population prevalence. Population size was defined based on data from the 2012 Brazilian Registry of Transplant (RBT 2012) available through the Brazilian Association of Transplantation [49]. Of the 57,815 patients who received a KT in Brazil from 2000 to 2012, we only considered the 31,241 patients registered to be under follow up in RBT 2012 as eligible for our calculation. Based on a non-adherence prevalence rate of 50%, the 95% confidence interval with a 5% sample error, and a design effect of 3.0, a sample size of 1139 patients was calculated and, considering the multi-stage design of the study, divided into approximately 283 patients in each stratum.

The number of patients included by each centre, considered as clusters in the analysis, is proportional to the number of transplants performed per year and the number of patients in follow-up, so called the size of the KT centre. According to the RBT 2012, there were 123 active transplant centres in Brazil, distributed across 22 states, which performed 5385 KTs in that year [49]. Due to the absence of an official classification of KT centres based on their activity, we proposed the following classification, which is based on a pre-existing classification for heart transplantation [18], and taking into consideration the profile of the centres included in the study: low activity, ≤50 KTs/year; moderate activity, 50-150 KTs/year, and high activity, ≥150 KTs/year. We used the 5 years prior to the study period (2010 to 2014) as the reference period for the KT activity of a centre. Our classification was validated by consulting specialists through a web survey, with 92% of respondents considering our proposal as either ‘adequate’ or ‘very adequate’.

### Variables and measurements

The selection of variables was guided by the ecological model, shown in Fig. 1, which considers the influence of multiple levels of health care as potential determinants of a patient’s behaviour [9, 50]. Variables are measured using established instruments, investigator-developed measures specific for this study or collected from medical records. A detailed description of the instruments and operational definitions used are summarized in Additional file 1: Tables S1 and S2.

#### Behavioural factors

The implementation phase of medication adherence (taking and timing dimensions, drug holidays and dose reduction) [51] to immunosuppressive drugs is measured using three methods: a validated self-report (Basel Assessment of Adherence with Immunosuppressive Medication Scale - BAASIS) [28, 43], blood assay [52], and collateral reports by health care workers [53]. In addition to the evaluation provided by each individual method, a composite adherence score is calculated. Overall non-adherence to immunosuppressants will be defined by a positive finding on any one of the three assessment methods [27, 29]. For blood assay of immunosuppressants, the actual level and the levels in the two previous assays are being used [29, 46], as described in Additional file 1: Table S1.

The current physical activity level [54, 55], smoking status [18, 56], alcohol use [18, 57], and appointment keeping [18] are assessed by using investigator-developed questions based on previous transplant research. These questions are presented in Additional file 1: Table S1.

#### Multilevel correlates of non-adherence to immunosuppressive medication

##### Patient level

Demographic (age, sex, race, education, employment status, marital status, family income), disease related (aetiology of kidney disease, treatment modality prior to transplant, preemptive transplant, time on dialysis, donor type, post-transplant time, comorbidity, height, weight), and therapy related data (drugs of immunosuppressive schema, number of immunosuppressive medications, daily number of doses of immunosuppressants) are collected through structured interview and review of charts [20, 26, 58–61]. The following post-transplantation clinical data is collected from medical charts: number of treated acute rejection episodes, creatinine level, estimated glomerular filtration rate, and re-hospitalizations [52, 61–64] (Additional file 1: Table S2).

##### Health care provider level (Micro level)

A patient’s trust in the transplant team and satisfaction with his/her relationship with health care providers are evaluated using a visual analogue scale (VAS) [65]. Social support provided to the medication taking is also assessed by two questions [18] (Additional file 1: Table S2).

##### Health care organization level (Meso level)

With regard to CIM, we include questions on patient-centred care, continuity and coordination of care, information and communication technology, organization, and continuous education provided [18, 66, 67]. Demographic characteristics [20, 67, 68] and satisfaction with operational access to the transplant centre are also collected [14, 69]. All data are collected through a self-report questionnaire completed by a nurse or physician nominated by the transplant centre manager (Additional file 1: Table S2).

##### Healthcare system and policy level (Macro level)

For KT patients, the Brazilian Public Health System offers outpatient follow-up, immunosuppressive drugs, laboratory exams, and hospitalizations. As the public system is sometimes inefficient in providing access to the required exams and to hospitalization, some individuals do choose to pay for a complimentary private health insurance. Self-reported financial burden is assessed by identifying the complimentary private health insurance status [20]. City and region specific data related to health care access, such as number of hospitals and the number of intensive care beds available, and relevant information about the transplant centre are retrieved from the 2012 RBT [49], as summarized in Additional file 1: Table S2.

### Recruitment/data collection

Recruitment and data collection are centrally managed by the coordinator centre, the Federal University of Juiz de Fora, Juiz de Fora. After obtaining approval of the project by the ethics research board (ERB) of the coordinating centre, all invited centres submitted the project to their local ERB. All data collection is performed using the Research Electronic Data Capture (RedCap) system. Local research coordinators receive a detailed step-by-step training guide, followed by a specific training session via phone or podcast. Backup support for data collection is continuously available through the ADHERE BRAZIL study via phone, e-mail, or Skype. Once the project is locally approved by ERB and the local research team has been trained, the access to RedCap system is provided through a unique identification and password. This allows the centre to update and visualize only its data. The RedCap system is a safe internet program, created by Vanderbilt University, designed exclusively for the capture and storage of data that can accessed remotely by trained individuals. RedCap allows data to be collected, organized, and stored in an integrated manner, making the process of data analysis less time-consuming and feasible across multiple centres. Furthermore, users can access their database at any time to complete or update their data over the collecting time (http://www.project-redcap.org/).

#### Patient data collection

Data will be collected over an 18-month period, during regular medical visits to the transplant service and directly entered into the RedCap database by a trained professional. Scheduled patients are randomly selected using routine computerized method. Eligible patients, randomly selected by a list generated with the blockrand package for R language, are invited to participate and to sign the informed consent. Then, the BAASIS interview [28, 43] and the structured questionnaire are applied. The collateral report is completed by the physician/nurse on the same day. Blood levels of immunosuppressive drugs and other clinical variables are extracted from the medical records.

#### Transplant centre’s data collection

A representative of the transplant service, indicated by the centre’s manager, enters the data from the questionnaire directly into Red Cap system.

Data collection began on December 7, 2015, and is planned for 18 months, with expected completion in June 2017. The quality of the information registered into the RedCap system of each centre is verified by the central study coordinators on a weekly basis, ensuring that there is no missing data or errors in data entry, and reports of found inadequacies are regularly, once a week, reported to each centre. Once data collection is completed, a summary report of adherence rates and factors associated with non-adherence will be provided to each centre. After completion of the study, a benchmark report will be sent to each transplant centre, in which the results of each will be summarized and compared to the data from other centres. Only the information of the centre receiving the report will be individualized, with the data from other centres presented in pooled and anonymized manner.

### Statistical analysis plan

#### Aim 1

In addition to the general prevalence estimates of non-adherence to health behaviours, the adopted sample design allows for stratified estimates to be calculated according to region (South/Southeast, North/Midwest/Northeast), the activity level of the transplant centre, and the sample strata. Prevalence estimates will be presented as percentages, with their respective 95% confidence intervals (95% CI), calculated using Taylors’ Linearization Series given the multistage sampling design of the study. For numerical variables with a normal distribution, the mean and respective 95% CI will be calculated and for variables with significantly skewed distribution, the median and inter-quartile interval. Box-plots and normal probability plots will be used to assess the distribution properties. Graphics will be used, where relevant, to enhance visualization, including grouped box-plots and bar plots for prevalence.

#### Aim 2

To explore the association between medication non-adherence and multilevel studied correlates, the analyses will follow the multilevel ecological model, following the hierarchical approach [70]. This approach is a forward driven variable selection method, based on the epidemiological framework adopted, following the proximity of the variable levels to the patient in a theoretical causal framework. The non-adherence indicator (binary) variable will be analysed using generalized estimation equations (GEE). This population average approach is an adequate modelling framework that can handle the multistage nature of the study design, as well as provides flexibility regarding the probability distribution adequate to each outcome variable nature, and consequently the association parameter to be estimated. For instance, binary outcomes, such as non-adherence, will be modelled using the Poisson distribution and the log link function, providing prevalence ratios as association parameter. Discrete or count outcomes, like absence at scheduled follow-up consultations, can also be modelled as a Poisson or Negative Binomial distribution with log link function, but providing a counting rate ratios as the association parameter. Association effects will be considered of scientific relevance on the basis on their magnitude (effect size), precision (by their 95% CI) and associated *p*-value. As per the recent statement from the American Statistical Association, terms such as ‘statistically significant and model adjustment based only on p-values’ will no longer be adopted in the analysis [71].

All analyses will be performed using IBM Statistics (SPSS 24.0, Chicago, IL, USA) and STATA (version 14, StataCorp LP, College Station, TX, USA) statistical packages. All codes used will be publically available, permitting reproducibility.

### Ethical considerations

The study was reviewed and approved by the ERB of University Hospital of Federal University of Juiz de Fora (691.120), and nationally registered (CAAE 27972914.1.1001.5133). All participating centres also locally submitted the protocol to ERB approval before data collection. Information and informed consent followed the guidelines of the Declaration of Helsinki (World Medical Organization 1996) and specific national legislations. Written informed consent will be obtained from all the participants before the data collection.

## Discussion

By using appropriate methodology, our study will provide a broad and more representative information regarding non-adherence among KT patients in Brazil. Furthermore, we will also identify factors associated with medication adherence, especially at the level of health care service and health care team, which could provide potential leverage points for developing further interventions. This is the first multi-centre study that is assessing adherence and multilevel correlates in KT patients and in Brazil, which has a universal and public health care system and a territorial varsity. The social and economic diversity across the country, which mirrors the variation in the availability and access to health care, offers a unique opportunity to explore non-adherence. It is one of the few studies assessing patient non-adherence to the different aspects of the post-KT treatment, including: medication intake, alcohol and smoking cessation, physical activity and appointment keeping in a large KT population.

The use of a consistent methodology of non-adherence to medication measurements will provide an accurate prevalence and allow us to conduct reliable comparisons between centres, regions, and clinical practice patterns. Our sampling methodology tried to avoid selection bias of patients, given that the centres were selected by convenience. Recruiting centres from all regions, and then including different patterns of clinical practice, administration and transplant activity, and a randomized selection of patients, will increase the reliability of the study findings to Brazil and to other countries with similar health systems. The multilevel analysis of factors associated with non-adherence, including at the level of the patient, healthcare provider, health care organization, health care system and policies, will enable us to capture a wide range of factors correlated to non-adherence. Furthermore, we believe that the identification of the clinical practice patterns positively associated with adherence might be the most valuable potential contribution of this study, and such practices can be promptly adopted in KT care. This information, for the first time involving a large sample of KT patients, will provide an accurate view of the current state of non-adherence after KT, and identify targets for further interventions. Data collection using the RedCap system promotes a constant and ongoing quality surveillance of included data. The RedCap system provides a useful tool for collecting data across multiple centres and for problem solving, at a distance, which is essential considering that our study covers a territory of 328,804 mi^2^ and 200 million inhabitants. As another relevant point, we will provide feedback to each centre regarding their patients’ adherence behaviours, which the centres can use to improve their clinical practice.

Our study has certain limitations. First, although the 20 centres included in the study were representative of different kinds of centres and regions of Brazil, these centres were selected and recruited by convenience, which allowed our study to be economically feasible and to achieve a potential low dropout rate. Although centres were selected by convenience, patients were randomly selected. Second, due to limited resources, it was not possible to evaluate non-adherence to immunosuppressants using electronic monitoring. We opted to apply a combination of diagnostic methods [27], (i.e. patient self-report, collateral report from nurse/physician, and immunosuppressive blood levels) which has previously been shown to provide the highest sensitivity compared to electronic monitoring [29]. Our evaluation of the CIM model is limited but, to our knowledge, this will be the first time that this model is being evaluated within the context of KT. Finally, as a cross-sectional study, causality between identified factors and non-adherence to post-KT treatment cannot be inferred.

In conclusion, the ADHERE Brazil study is a cross-sectional study evaluating various aspects of non-adherence behaviour to the treatment regimen, and multi-level correlates to immunosuppressive medication non-adherence in 1130 patients across 20 KT centres. Data from this study will provide evidence to people involved in treating KT patients (health care professionals, health care policy makers) regarding the level (patient, health care professional, and health care system and policies) at which further interventions should be implemented to improve adherence to the post-KT treatment and, consequently, the long-term outcomes.

## Additional file

Additional file 1: **Table S1.** Behavioural factors. **Table S2.** Multilevel correlates of nonadherence to immunosuppressants. (DOCX 50 kb)

## Abbreviations

BAASIS: The Basel assessment of adherence with immunosuppressive medications scale
CIM: Chronic illness management
KT: Kidney transplant
