# Supplementary material for: Prevalence and correlates of non-adherence to immunosuppressants and to health behaviours in patients after kidney transplantation in Brazil – the ADHERE BRAZIL multicentre study: a cross-sectional study protocol
Source: BMC Nephrol. 2018 Feb 20;19:41. doi: 10.1186/s12882-018-0840-6 (PMC5819659; doi:10.1186/s12882-018-0840-6)
Supplement: Supplementary file 1 — Table S1. Behavioural factors. Table S2. Multilevel correlates of nonadherence to immunosuppressants. (DOCX 50 kb) [file 12882_2018_840_MOESM1_ESM.docx]

**Table S1:** Behavioural factors

| **Variable** | **Instrument** | - **# of items** - **Recall period** - **Response options** - **Scoring** | **Validity/Reliability** |
| --- | --- | --- | --- |
| Self-reported non-adherence to  to immunosuppressive medications  (Structured interview) | Basel Assessment of Adherence with Immunosuppressive Medications Scale® (BAASIS) [1,2] Implementation (taking dimension; drug holiday; timing dimension; dose reduction) | - 4 items - 4 weeks - Yes/No - Any ‘yes’ answer is considered ‘non-adherence’ to the item. Non-adherence = any taking non-adherence, timing non-adherence, drug holiday, and dose reduction. | **Concurrent validity** assessed in kidney transplant recipients [2].  **Predictive validity** assessed in liver transplant recipients [3] |
| Patient’s immunosuppressive medication non- adherence rated by nurse and physician (collateral report)  (Written self-report) | Item from MAESTRO study [4] | - 1 item - 4 weeks - Patients’ adherence rated by nurse and physician on 3-point Likert scale ranging from ‘1= excellent’ to ‘3= poor’ - Non-adherence = any rating other than ‘excellent’ |  |
| Blood level (extracted from patient´s file) | [4,5] | - 3 measurements - Actual levels and levels on the two previous measurements - Below therapeutic levels, defined by the transplant team - Yes/no |  |
| Level of physical activity  (Structured interview) | Adapted from Brief Physical Activity Assessment tool [6,7] | - 2 items - Questions  1. Do you perform any physical activity, Yes/No 2. If yes, # min/week   Sufficiently active: ≥ 150 min/week of at least moderate physical activity. Non-adherent = not being sufficiently active | Assessed against accelerometer reasonable  - **criterion validity** (ĸ= 0.40, 95% CI= 0.12-0.69) [6]  - **moderate inter-rater reliability** (ĸ= 0.53, 95% CI= 0.33-0.72) [6] |
| Smoking status  (Structured interview) | [8,9] | - 1 item - 1 year recall period - 4-point Likert scale;   1 = yes to 4 = no, I never smoked.   - Non-adherence= patients that reported to currently smoke. | No further information on psychometric properties is currently available. |
| Alcohol use  (Structured interview) | [9,10] | - 2 items, - 1 week/1 year recall period   Yes/no;  Moderate drinker: 1 drink/day (women), 2 drinks/day (men); Heavy drinker: >1 drink/day, >2 drinks/day (men)   - Non-adherent = heavy drinkers | No further information on psychometric properties is currently available. |
| Non-adherence to appointment keeping  (Structured interview) | [10] | - 1 item - Last 5 appointments recall period - 5-point Likert scale ranging from ‘1= none’ to ‘6= all’ - Non-adherent = missed ≥ 1 appointment | No further information on psychometric properties is currently available. |

**Table S2.** Multilevel correlates of nonadherence to immunosuppressants.

| **Variable** | **Instrument** | - **# of items** - **Recall period** - **Response options** - **Scoring** | **Description** |
| --- | --- | --- | --- |
| **Patient Level** | | | |
| Demographics  (Structured interview) | Investigator-developed self-report questionnaire, based on previous transplant research [11-15] | - 9 items | Date of birth, gender, race, marital status, education, employment, family income, religion |
| Condition-related factors  (Structured interview) | Investigator-developed self-report questionnaire, based on previous transplant research [11-15] | - 8 items | Aetiology of kidney disease, treatment modality previous to transplant, pre-emptive transplant, time on dialysis, donor type, and post-transplant time, obesity, comorbidities  (Chart review) |
| Therapy-related factors  (Structured interview) | Investigator-developed self-report questionnaire, based on previous transplant research [13,16] | - 2 items | Drugs of immunosuppressive schema, number of prescribed immunosuppressive medications, and the daily number of dosing times of immunosuppressants |
| Post transplantation clinical data | Investigator-developed self-report questionnaire, based on previous transplant research [5,14,15,17-19] | - 4 items | Number of treated acute rejection episodes, creatinine, estimated glomerular filtration rate, re-hospitalizations. |
| **Health Care Provider Level** | | | |
| Patient satisfaction with the interpersonal dimension of care  (Structured interview) | VAS scale [20] | - 1 items - Current situation - Ranging from ‘0 = not satisfied’ to ’100 = very satisfied’ |  |
| Trust in transplant team  (Structured interview) | VAS scale [20] | - 1 items - Current situation - Ranging from ‘0 = not confident’ to ’100 = very confident’ |  |
| Received social support  (Structured interview) | Investigator-developed questions based on previous transplant research [9] | - 2 items - Current situation | Received help from a third person for taking medication, tips |
| **Health Care Organization Level** | | | |
| Core competencies of KT team in view of taking care of chronically ill patients  (Written self-report) | Investigator-developed based on previous transplant research [21,22] | - 10 items to assess core competencies | 1) Patient-centred care (2 items)  2) Continuity and coordination (3 items)  3) Information and communication technology (1 item)  4) Organization and continuous education (5 items) |
|  |  |  |  |
| Structural characteristics of the transplant centre  (Written self-report) | Investigator-developed based on previous research [21,23,24] | - 13 items | Number of beds, transplant activity (number of KT/year), number of KT last 5 years, patients on follow-up, multi-professional team, composition of multi-professional team, number of physicians, number of nurses, type of centre (public, private, public/private), teaching centre, teaching activities (graduate and post graduate programs) |
|  |  |  |  |
| Satisfaction with access to the transplant centre (Written self-report) | Investigator-developed based on previous research [25,26] | - 7 items | Access by transportation, internal access within an institution, appointment scheduling system, frequency of consultation, physical structure |
| **Healthcare system and policy level** | | | |
| Perceived financial burden of treatment  (Structured interview) | Investigator-developed based on previous research [13,24] | - 2 items   Private complimentary insurance coverage:   - Current status - Yes/No   Lab exams by private complimentary insurance coverage:   - Current status - 3-point Likert scale ranging from ‘1 =   Yes, fully’ to ‘3 = No’ | Private complimentary insurance coverage available |
| Mechanism for immunosuppressants refill  (Structured interview) | Investigator-developed based on previous research [27] | - 2 items   Location (city) for refilling of prescribed immunosuppessants  Distance to the location |  |
| City/region related data  (Collected from RBT, 2015) |  | - City, Brazilian region - Number of KT services, number of KT performed in last 5 years - Number of hospitals, number of intensive care beds - Presence/absence of a medical school |  |

KT, kidney transplantation; RBT, Brazilian Registry of Transplant

**References**

1. Dobbels F, Berben L, De Geest S, Drent G, Lennerling A, Whittaker C, et al: **The psychometric properties and practicability of self- report instruments to identify medication nonadherence in adult transplant patients: a systematic review.** *Transplantation* 2010, **90**(2):205-19.
2. Marsicano EO, Fernandes NS, Colugnati F, Grincenkov FR, Fernandes NM, De Geest S, et al: **Transcultural adaptation and initial validation of Brazilian-Portuguese version of the Basel assessment of adherence to immunosuppressive medications scale (BAASIS) in kidney transplants.** *BMC Nephrol*. 2013:**14**: 3-8.
3. Ducci J, De Simone P, Denhaerynck K, Dobbels F, De Geest S: **Correlates of subclinical non adherence to immunosuppression after liver transplantation.** *Transpl Int.* 2013,**26**(Suppl 2), 99.
4. Schäfer-Keller P, Steiger J, Bock A, Denhaerynck K, De Geest S: **Diagnostic accuracy of measurement methods to assess non-adherence to immunosuppressive drugs in kidney transplant recipients.** *Am J Transplant*. 2008, **8**(3):616–26.
5. Denhaerynck K, Burkhalter F, Schäfer-Keller P, Steiger J, Bock A, De Geest S: **Clinical consequences of non adherence to immunosuppressive medication in kidney transplant patients**. *Transpl Int*. 2009,**22**(4):441–46.
6. Marshall, A.L., Smith, B.J., Bauman, A.E. & Kaur, S: **Reliability and validity of a brief physical activity assessment for use by family doctors.** *Br J Sports Med*. 2005, **39**(5):294-97.
7. World Health Organization. **Global recommendations on physical activity for health.** Genebra: WHO; 2010. Available: http://whqlibdoc.who.int/publications/2010/9789241599979_eng.pdf. Access on August 30th 2016.
8. Fiore M; Jaen CR; Baker TB. **Treating Tobacco Use and Dependence: 2008 Update**. May 2008 ed. Rockville, MD: U.S. Department of Health and Human Services.
9. Berben L, Denhaerynck K, Dobbels F, Engberg S, Vanhaecke J, Crespo-Leiro MG, et al; BRIGHT Study consortium: **Building research initiative group: chronic illness management and adherence in transplantation (BRIGHT) study: study protocol**. *J Adv Nurs*. 2015, **71**(3):642-54.
10. World Health Organization – WHO. **Global status report on alcohol**. Genebra: WHO, 2004.
11. Russell, C.L., Kilburn, E., Conn, V.S., Libbus, M.K. & Ashbaugh, C: **Medication-taking beliefs of adult renal transplant recipients**. *Clin Nurse Spec.* 2003, **17**(4):200-8/209-30.
12. Butler, J.A., Peveler, R.C., Roderick, P., Smith, P.W., Horne, R. & Mason, J.C: ; **Modifiable risk factors for non-adherence to immunosuppressants in renal transplant recipients: a cross-sectional study.** *Nephrol Dial Transplant*. 2004, **19**(12):3144-9.
13. Denhaerynck, K. Steiger J, Bock A, Schäfer-Keller P, Köfer S, Thannberger N, et al: **Prevalence and risk factors of non-adherence with immunosuppressive medication in kidney transplant patients.** *Am J Transplant*. 2007,**7**(1):108-16.
14. Spivey CA, Chisholm-Burns MA, Damadzadeh B, Billheimer D: **Determining the effect of immunosuppressant adherence on graft**

**failure risk among renal transplant recipients.** *Clin Transplant*.2014,**28**(1):96-104.

1. Prihodova L, Nagyova I, Rosenberger J, Majernikova M, Roland R, Groothoff JW, et al: **Adherence in patients in the first year after kidney transplantation and its impact on graft loss and mortality: a cross-sectional and prospective study.** *J Adv Nurs*. 2014, **70**(12):2871-83.
2. Fine RN, Becker Y, De Geest S, Eisen H, Ettenger R, Evans R, et al: **Nonadherence consensus conference summary report**. *Am J Transplant*. 2009,**9**(1):35-41.
3. Vlaminck H, Maes B, Evers G, Verbeke G, Lerut E, Van Damme B, et al: **Prospective study on late consequences of subclinical non-compliance with immunosuppressive therapy in renal transplant patients.** *Am J Transplant*. 2004,**4**(9):1509-13.
4. Nevins TE, Thomas W: **Quantitative patterns of azathioprine adherence after renal transplantation.** *Transplantation.* 2009,**87**(5):711-18.
5. Nevins TE, Robiner WN, Thomas W: **Predictive patterns of early medication adherence in renal transplantation.** *Transplantation*. 2014, **98**(8):878-84.
6. Voutilainen A, Itk Aaho PT, Kvist T, Vehvil AJK: **How to ask about patient satisfaction? The visual analogue scale is less vulnerable to confounding factors and ceiling effect than a symmetric Likert scale.** *J Adv Nurs.* 2016,**72**(4):946–57.
7. Berben L, Russell CL, Engberg S, Dobbels F , De Geest S**: Development, content validity and inter-rater reliability testing of the Chronic Illness Management Implementation – Building Research Initiative Group: Chronic Illness Management and Adherence in Transplantation: An instrument to assess the level of chronic illness management implemented in solid organ transplant programmes.** *Intern J Care*. 2014,**17**(1–2):59–71.
8. World Health Organization. **Preparing a health care workforce for the 21st century: the challenge of chronic conditions**. World Health Organization, Geneva, 2005.
9. Bissonnette J, K. Woodend, B Davies, D Stacey, GA Knoll: **Evaluation of a collaborative chronic care approach to improve outcomes in kidney transplant recipient.** *Clin Transplant* 2013, **27**: 232–238.
10. Marsicano EO, Fernandes NS, Colugnati FA, Fernandes NM, De Geest S, Sanders-Pinheiro H: **Multilevel Correlates of Non-Adherence in Kidney Transplant Patients Benefitting from Full Cost Coverage for Immunosuppressives: A Cross-Sectional Study**. *PLoS One.* 2015,**30:** 10(11).
11. Melchior R, Nemes MI, Basso CR, Castanheira ER, Alves MT, Buchalla CM, et al: **Evaluation of the organizational structure of HIV/AIDS outpatient care in Brazil.** *Rev Saude Publica* 2006, **40**(1):143-151.
12. BRASIL, Ministério da Saúde. Secretaria de Vigilância em Saúde. Programa Nacional de DST e Aids. **AVALIAÇÃO DA QUALIDADE DA ASSISTÊNCIA AMBUATORIAL NOS SERVIÇOS PÚBLICOS DE ATENÇÃO À AIDS NO BRASIL - Sistema de Avaliação Qualiaids**. Departamento de DST, Aids e Hepatites Virais, Equipe de Pesquisa Qualiaids, Departamento de Medicina Preventiva, Faculdade de Medicina, Universidade de São Paulo. 2006. Available: http://www.qualiaids.fm.usp.br/Access on August 30th 2016.
13. Foster BJ, Pai A, Zhao H, Furth S, TAKE-IT Study Group: **TAKE-IT study: aims, design, and methods**. *BMC Nephrol*. 2014, **30**(15):139.
